# Identification and expression of functionally conserved circadian clock genes in lichen-forming fungi

**DOI:** 10.1038/s41598-022-19646-y

**Published:** 2022-09-23

**Authors:** Henrique F. Valim, Francesco Dal Grande, Jürgen Otte, Garima Singh, Dominik Merges, Imke Schmitt

**Affiliations:** 1 Senckenberg Biodiversity and Climate Research Centre (BiK-F), Senckenberganlage 25, 60325 Frankfurt am Main, Germany; 2 LOEWE Center for Translational Biodiversity Genomics (TBG), Senckenberganlage 25, 60325 Frankfurt am Main, Germany; 3 Department of Biology, University of Padua, Via U. Bassi 58/B, Padua, Italy; 4 Department of Forest Mycology and Plant Pathology, Swedish University of Agricultural Sciences, P.O. Box 7070, 750 07 Uppsala, Sweden; 5 Department of Biological Sciences, Institute of Ecology, Evolution and Diversity, Goethe University Frankfurt, Max-von-Laue-Straße 13, 60438 Frankfurt am Main, Germany

**Keywords:** Evolutionary biology, Gene expression, Functional genomics, Phylogeny, Protein function predictions, Phylogenetics, Reverse transcription polymerase chain reaction

## Abstract

Lichen-forming fungi establish stable symbioses with green algae or cyanobacteria. Many species have broad distributions, both in geographic and ecological space, making them ideal subjects to study organism-environment interactions. However, little is known about the specific mechanisms that contribute to environmental adaptation in lichen-forming fungi. The circadian clock provides a well-described mechanism that contributes to regional adaptation across a variety of species, including fungi. Here, we identify the putative circadian clock components in phylogenetically divergent lichen-forming fungi. The core circadian genes (*frq, wc-1, wc-2, frh*) are present across the Fungi, including 31 lichen-forming species, and their evolutionary trajectories mirror overall fungal evolution. Comparative analyses of the clock genes indicate conserved domain architecture among lichen- and non-lichen-forming taxa. We used RT-qPCR to examine the core circadian loop of two unrelated lichen-forming fungi, *Umbilicaria pustulata* (Lecanoromycetes) and *Dermatocarpon miniatum* (Eurotiomycetes), to determine that the putative *frq* gene is activated in a light-dependent manner similar to the model fungus *Neurospora crassa*. Together, these results demonstrate that lichen-forming fungi retain functional light-responsive mechanisms, including a functioning circadian clock. Our findings provide a stepping stone into investigating the circadian clock in the lichen symbiosis, e.g. its role in adaptation, and in synchronizing the symbiotic interaction.

## Introduction

The circadian clock is a well-described central molecular timekeeping mechanism that regulates biochemical and physiological processes within most organisms, helping them to perceive and respond to abiotic and biotic environmental cues. Circadian systems are characterized by oscillations that can be synchronized (entrained) by environmental cues (i.e. *zeitgebers*) such as light, heat, or nutrients^1^. The entrained circadian rhythms can persist without external cues with a period of roughly 24 h under free-running conditions and are capable of temperature compensation, wherein the circadian clock maintains stable rhythmicity across broad temperature ranges within which biochemical and physiological processes are regulated^2^.

The central circadian oscillator of *N. crassa* is composed of the negative element frequency (*frq*) and its interactions with the White Collar Complex (WCC), composed of proteins encoded by the white collar-1 (*wc-1*) and white collar-2 (*wc-2*) genes. WCC activates transcription of *frq,* which ultimately represses its own expression by affecting the phosphorylation of WC-1 and WC-2^3,4^. Another essential component of the circadian regulator is Frequency Interacting RNA Helicase (FRH), which plays a role in regulating FRQ expression by variably protecting FRQ against ubiquitin-mediated degradation and suppressing *frq* expression via interaction with the WCC^5^. This central clock oscillator in turn regulates a variety of downstream transcriptional and post-translational modifications^6^. The *N. crassa* circadian clock also exhibits a temperature compensation mechanism, consistent with other circadian clock systems in plants and animals^7,8^.

Circadian systems are present in nearly all organisms across the various kingdoms of life, and appear to have arisen at least three times during the evolution of life^9–11^. The adaptive value of the circadian system becomes obvious in populations that have lost circadian rhythms over the course of their evolutionary history, or that “switch off” their circadian clocks due to seasonal cues; in both cases, the circadian system becomes less rhythmic under conditions where rhythmicity does not provide adaptive information to the biological system. Independently-evolved populations of the cavefish *Astyanax mexicanus* show widespread disruption of circadian clock gene rhythmicity, as well as a reduction in rhythmic transcription compared to surface-dwelling populations^12^. Activating overwintering mechanisms in trees involves an interplay between photoreceptors and the circadian clock, which is then disrupted for the duration of the winter^13^. The circadian rhythms of Svalbard reindeer are attenuated during winter months^14^, similarly to that of other Arctic mammals such as the muskox *Ovibos moschatus*^15^ and the red fox *Vulpes vulpes*^16^.

The circadian clock provides fitness and performance advantages in a variety of model systems, further supporting its role in environmental adaptation. In plants, the circadian clock aligns chemical defenses to herbivore feeding patterns, regulates drought responses, and anticipates pathogen attack (see Xu et al. 2022^17^ for a recent review of the role of the circadian clock in plant biotic and abiotic stress responses). In fungi, the asexual reproductive patterns of *Neurospora crassa* have long been observed to be regulated by the circadian clock^18,19^, while strains of *Neurospora discreta* with habitat-specific circadian rhythms maintain higher fitness in their respective habitats^20^.The interacting circadian clocks in symbiotic systems are a growing topic of interest^21,22^. Tightly-regulated circadian systems have been observed between corals and algae^23,24^, as well as between the Hawaiian bobtail squid *Euprymna scolopes* and the bioluminescent bacteria *Vibrio fischeri*^25^. The arbuscular mycorrhizal fungus *Rhizoglomus irregularis* also contains a functioning circadian clock system^26^, which has been hypothesized to play a role in the AMF-plant symbiosis.

The lichen symbiosis is composed of a fungal partner (mycobiont) and a photosynthesizing partner, either a green alga and/or a cyanobacterium (photobiont), plus a more or less specific suite of associated prokaryotic and eukaryotic microorganisms (e.g.^27,28^). The lichen lifestyle—a fungal nutritional mode that relies on the photosynthetic products of internally accommodated algal symbionts—occurs in unrelated lineages across the fungal tree of life, but is most prevalent in the Leotiomyceta (e.g. Lecanoromycetes, Eurotiomycetes, Dothideomycetes) within the Ascomycota^29^. Lichens are ubiquitous in the landscape, thriving in a diverse range of habitats across nearly all ecosystems^30^. This ubiquity is likely related to lichens’ capability to withstand extreme abiotic stresses, such as complete desiccation^31,32^. This ability to withstand stress may be due to variable stress response pathways relative to other sessile organisms; the lichen *Endocarpon pustillum* maintains active transcription of metabolism-associated genes during osmotic stress that are largely suppressed in plants and fungi^33^.

Although lichens have been acknowledged as a symbiosis of interest to explore symbiotic circadian clock systems^22,34^, little work has been done to elucidate the circadian clock mechanism in lichens aside from the identification of the putative *frq* ortholog in the lichen-forming fungus *Umbilicaria pustulata*^35^. An important prerequisite step to further studies of the lichen circadian clock is to determine how conserved the core circadian clock and photosensory machinery is across phylogenetically unrelated lichen-forming fungi, as well as to determine whether this core machinery is functional.

Here, we investigate the presence of putative circadian clock components across the Fungi, focusing on major lineages in the Ascomycota that include lichens. We performed this investigation using a two-pronged approach: a phylogenetic analysis of putative homologs of the core circadian clock genes *frq, wc-1, wc-2,* and *frh,* and functional validation of one core mechanism in the fungal circadian clock. We find that homologs of these four core fungal circadian clock genes are present in lichen-forming Lecanoromycetes, Eurotiomycetes and Dothideomycetes, and that these orthologs contain strongly conserved protein-coding sequences in the functional domains of these genes. We demonstrate the light-dependent expression of the core clock gene *frq* in two highly diverged lichen-forming lineages, *U. pustulata* and *D. miniatum*. Taken together, these results demonstrate that lichen mycobionts retain functional light-responsive mechanisms, including a functioning circadian clock, similar to those of non-lichen-forming filamentous fungi.

## Materials and methods

### Identification and phylogenetic analysis of putative circadian clock genes

The four core fungal clock genes *frq, wc-1, wc-2,* and *frh* of the model fungus *Neurospora crassa* were used as queries to search for homologs in the genomes of the lichen-forming and non-lichen-forming fungi via reciprocal BLAST in GenBank, through the Genome Portal of the Joint Genome Institute^36^ and from Calchera et al*.*^37^; see Table S1.

Putative clock genes recovered from a broad range of fungal taxa across the Ascomycota, Basidiomycota, and Mucoromycota were used for the phylogenetic analysis. We made sure to include lichenized (and if possible non-lichenized) representatives of most Leotiomycete lineages that include lichens (Lecanoromyctes, Eurotiomycetes, Dothideomycetes). Amino acid sequence alignments were performed using *MAFFT* v7.450^38^. After alignment, sequences were trimmed using *TrimAl* v1.2^39^, removing all columns with gaps in more than 20% of sequences in the alignment. Phylogenetic trees were inferred by maximum likelihood using *RAxML* version 8^40^ using the GAMMA BLOSUM62 model and 1,000 bootstrap replicates. The presence of functional domains between lichen-forming and non-lichen-forming lineages was investigated using the protein domain and visualization tool DomainViz^41^.

### Study site and sample collection

Lichen thalli of *Dermatocarpon miniatum* and *Umbilicaria pustulata* were harvested from co-localized (ca. 600 m apart) sites in the vicinity of Eppstein, in the Taunus region of Germany (50°08′N 08°24′E). Single thallus fragments were taken from multiple populations (4 populations of *U. pustulata* and 5 populations of *D.* miniatum) to minimize disturbance. *U. pustulata* populations were located on horizontal or gently sloping, sun-exposed rock faces. *D. miniatum* populations were located on vertical, well-shaded rock faces. Samples were collected and briefly cleaned of debris before being transferred to labeled paper bags before immediate transfer to a growth chamber.

### Growth conditions and light exposure treatment

After sample collection, lichen thalli were radially divided into two pieces and placed in individually-wrapped 5 cm-diameter Petri dishes with sterile blotting paper and ca. 200 μl of sterile deionized water. Petri dishes were subsequently incubated for 72 h in a plant growth chamber (CLF Plant Climatics GmbH, Wertingen, DE) at 16 °C under a light:dark 12:12 h light regime with 30 μmol m^−2^ s^−1^ of light being provided during the light phase. After 72 h, all Petri dishes were wrapped in aluminum foil and placed in an additional paper box to induce a 24 h dark incubation period. Shortly before 8:00 (dark:dark 24 = circadian time 0, subjective dawn), control thalli were harvested into liquid nitrogen. Aluminum foil was then removed and light-treated Petri dishes were exposed for 20 min. Petri dishes were kept equidistant from light sources in the growth chamber during light exposure before being harvested for RNA extraction.

### Transcript abundance

After tissue harvesting from lichen thalli (150 mg), RNA was extracted with TRI Reagent (Zymo Research Europe GmbH, Freiburg, DE) according to the manufacturer’s instructions. Total RNA was quantified using a NanoPhotometer P 300 (Implen GmbH, Munich, DE) and cDNA was synthesized from 500 ng of total RNA using RevertAid H-Minus first-strand cDNA synthesis kit (MBI Fermentas) according to the supplied protocol. Reverse transcription quantitative PCR (RT-qPCR) was performed in an CFX Opus 96 cycler (Bio-Rad Laboratories GmbH, Feldkirchen, DE) using the Luna Universal qPCR Master Mix kit (New England Biolabs GmbH, Frankfurt am Main, DE). *Umbilicaria pustulata* and *Dermatocarpon miniatum actin-like 6A* was used as a reference gene.

### Statistical analyses

All data were analyzed using *R* v.3.4.2^42^ and *RStudio* v.1.0.153^43^. Pairwise *post-hoc* comparisons were made using the R package *EMMEANS*^44^ using Šidàk-adjusted contrasts after significant results were observed in a two-way ANOVA.

## Results

### Homologs of core circadian clock genes are present and highly conserved across lichen-forming fungal lineages

In order to investigate how widespread circadian clock-associated genes are across fungal lineages, we recovered putative homologs from the core circadian clock loop (*frq, wc-1, wc-2, frh*) across a wide set of lichen-forming and non-lichen-forming fungi (see Table S1). Putative homologs were included in the phylogenetic analysis after reciprocal BLAST using *Neurospora crassa* protein sequences.

**Figure 1:**
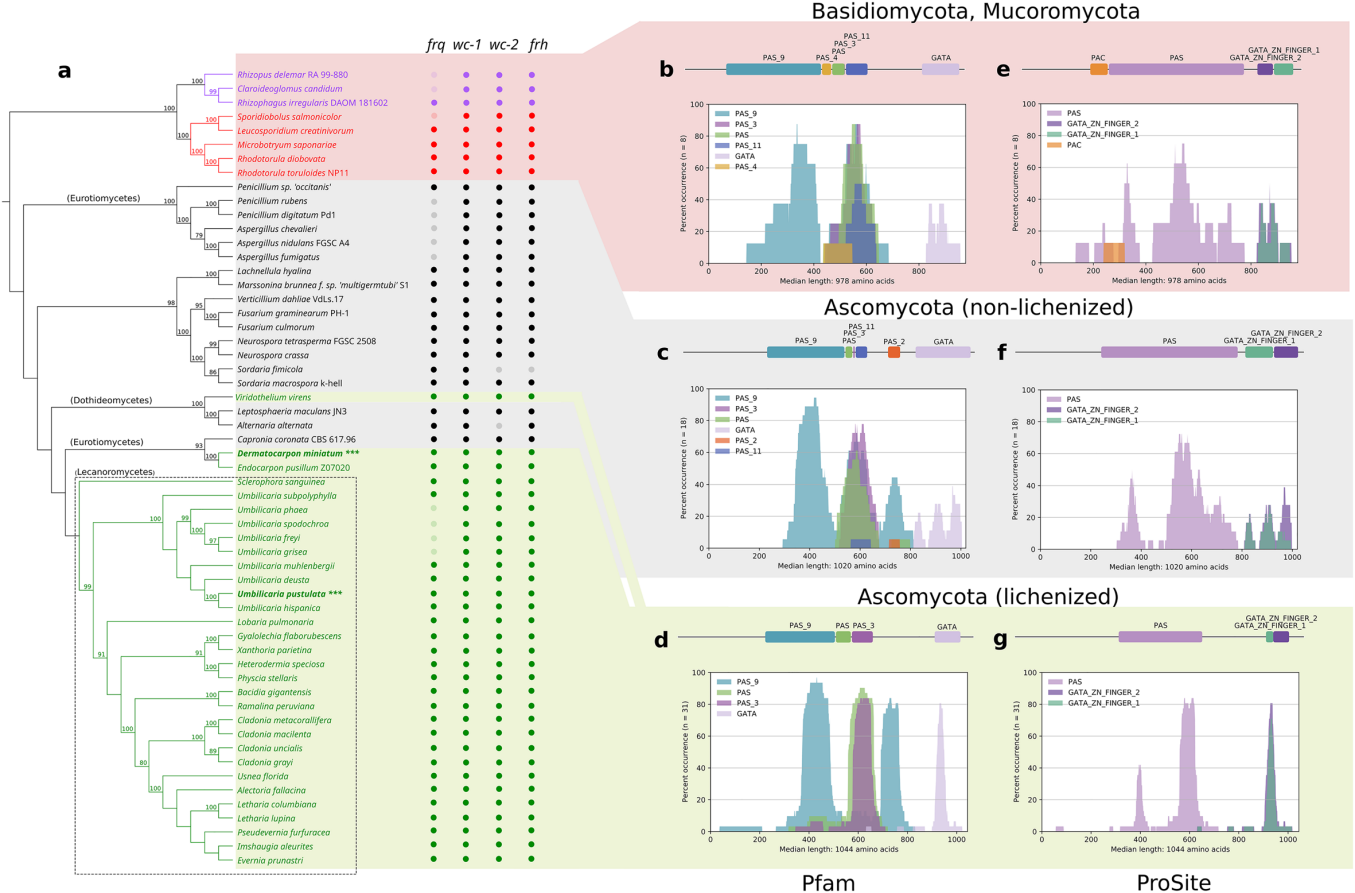
Presence of core circadian clock genes in fungi and domain architecture of White Collar-1 (wc-1). (**a**) Phylogenetic tree based on a WC-1 sequence alignment of 31 lichen-forming fungi (green) and a selection of non-lichen-forming taxa. This is a maximum likelihood tree. Bootstrap support greater than 75% is indicated at the branches. Taxa used in the experimental part of this study are marked by asterisks (***). Presence/absence of putative circadian homologs from *Neurospora crassa* is shown adjacent to each species. Functional domains annotated according to Pfam (**b**–**d**) and PROSITE (**e**–**g**) databases in lichen-forming (**d**, **g**) and non-lichen-forming lineages in the Ascomycota (**c**, **f**) as well as the Basidiomycota and Mucoromycota (**b**, **e**).

Putative homologs for lichen-forming taxa from a variety of clades were recovered, ranging from the class Lecanoromycetes to the classes Dothideomycetes and Eurotiomycetes. In order to explore potential variation between lichen-forming and non-lichen-forming fungal lineages, non-lichen-forming lineages from these classes were included when available from GenBank. Given that circadian clock homologs have been identified in lineages such as *Rhizophagus irregularis* in the Mucoromycota^26^ and have been predicted by functional annotation in the Basidiomycota, we further included lineages in these clades in the phylogenetic analysis. Circadian homologs for *wc-1* and *wc-2*, as well as for *frq* and *frh*, are present across most lichen-forming and select non-lichen-forming fungal clades; nonetheless, some putative homologs are missing in some lineages, including notably *frq* in the Trichocomaceae (e.g. *Aspergillus* spp. and *Penicillium* spp.) within the Eurotiomycetes and in several *Umbilicaria* spp. in the lichen-forming Lecanoromycetes (Fig. 1).

### Domain architecture of putative core circadian clock homologs is broadly conserved across lichen-forming and non-lichen-forming taxa

The phylogeny inferred for circadian clock homologs of WC-1 demonstrates a broad consensus with overall fungal evolution, including the phylogenetic placement of the lichenized taxa (Fig. 1a). In order to investigate whether functional domains in the core fungal circadian clock proteins were conserved, we compared the presence of functional domains between lichen-forming and non-lichen-forming lineages in the Ascomycota as well as the Basidiomycota/Mucoromycota in the Pfam (Fig. 1b–d) and PROSITE (Fig. 1e–g) protein domain databases using the protein domain and visualization tool DomainViz^41^.

The evolution of *wc-1* (Fig. 1), *wc-2*, *frq* and *frh* (Figs. S1–S3) largely mirrors known class-level relationships of the fungal tree of life. Clock genes of lichen-forming taxa fall within the respective fungal classes (Lecanoromycetes, Eurotiomycetes, Dothideomycetes) with high support, rather than forming a monophyletic clade. This suggests that there are no shared signatures of lichenization in the analyzed clock genes.

The results demonstrate that two key regions of the *Neurospora* WC-1 protein are broadly conserved between lichen-forming Ascomycota, non-lichen-forming Ascomycota, and Basidiomycota/Mucoromycota lineages. The first of these is the PAS region, originally identified in the *Drosophila melanogaster* period clock (PER), Ah receptor nuclear translocator (ARNT) and single-minded (SIM) proteins, and which is involved in protein dimerization, light perception, light regulation and circadian rhythm regulation. At the C-terminal end, there is broad conservation across all three investigated lineages in the GATA-type zinc finger motif (Fig. 1b–g), involved in protein localization to a set of consensus sequences in the regulatory regions of many light- and clock-regulated genes^45^.

### Light-dependent response of the core circadian clock gene frequency is conserved in two highly diverged lichen-forming fungal lineages

**Figure 2:**
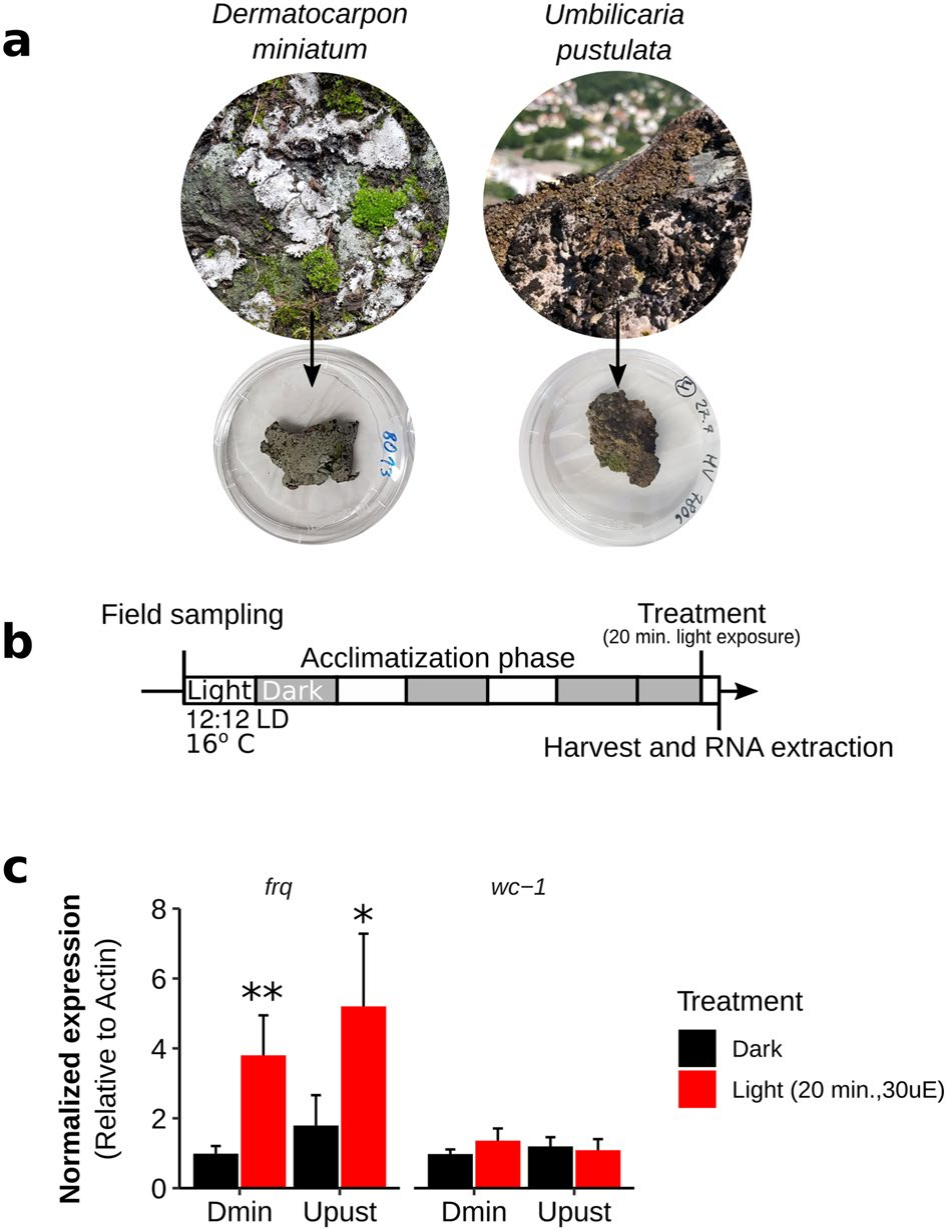
Homologs of *frq* in two lichen-forming fungi are responsive to light. (**a**) Thalli of the lichen-forming fungi *Dermatocarpon miniatum* and *Umbilicaria pustulata,* harvested in the Taunus mountain range of central Germany. (**b**) Lichen thalli were acclimatized to standard laboratory conditions via a 72 h LD 12:12 cycle at 16° C before 24 h in constant darkness, followed by a 20-min light induction at CT 0. (**c**) RT-qPCR analysis demonstrates broad conservation of light responsiveness in the circadian clock components of lichen-forming fungi. n = 6 for Dmin, n = 5 for Upust per treatment; mean ± SEM are shown. Asterisks represent significance extracted using Šidàk-adjusted contrasts: *P < 0.05; **P < 0.01; ***P < 0.001. Dmin: *Dermatocarpon miniatum*; Upust: *Umbilicaria pustulata*; *frq: frequency; wc-1: white collar-1.*

To investigate whether *frq* homologs in lichen-forming fungal lineages maintain their primary circadian function as targets of the WCC, we investigated the transcript abundance of *frq* putative homologs in response to light in *Dermatocarpon miniatum* (Ascomycota, Eurotiomycetes) and *Umbilicaria pustulata* (Ascomycota, Lecanoromycetes), two species belonging to different Ascomycete classes (Fig. 1a, asterisks). Thalli were harvested from a colocalized site in the Taunus mountains of Germany (Fig. 2a) and were acclimatized for 72 h under standardized conditions of 12:12 light:dark at 16° C, following which half of the thalli were exposed to 20 min of light (30 μE) before harvesting (Fig. 2b). All primers (Table S2) were previously tested for each species and no fluorescence was detected in negative controls (dH_2_0 and RT- controls).

After significant gene:treatment interactions were detected in ANOVA for both species, post-hoc tests revealed significantly increased *frq* transcript abundance in both *D. miniatum (p* = 0.0012, Šidàk-adjusted contrasts) and *U. pustulata (p* = 0.0101, Šidàk-adjusted contrasts) after exposure to light, while transcript abundance of *wc-1* was not significantly affected by the light treatment in either species (Fig. 2c). Statistical results for each species are summarized in Table S3.

## Discussion

The fungal circadian system, as elucidated initially in *Neurospora crassa,* is composed of three primary oscillators: a FRQ/WCC-dependent oscillator (FWO) as well as FRQ-independent (WC-FLO) and oscillators that are both FRQ- and WCC-independent (FLO)^46^.The main FRQ/WCC-dependent oscillator, of which *frq, wc-1, wc-2* and *frh* are the core components, was initially thought to be limited to Ascomycota, but has since been identified in various lineages across the Basidiomycota and Mucoromycota. Although a *frq* homolog has been previously identified in the genome of *Umbilicaria pustulata* within the lichen-forming Lecanoromycetes^35^, there have been no previous reports on the presence or absence of circadian clock genes across lichen-forming fungi more broadly. The identification of putative homologs of circadian and light-sensing genes in a wide range of lichen-forming fungal lineages, combined with the conserved light-activated function of the core circadian clock component *frq* in two highly diverged lichen-forming fungi, points to the conservation of the circadian clock mechanism in mycobionts. Lichen-forming fungi exist in stable symbioses with algae and cyanobacteria, both of which have relatively well-understood circadian clocks in free-living conditions^47–49^. The elucidation of the well-conserved fungal circadian mechanism in mycobionts thus opens the door for the investigation of how the circadian systems of myco- and photobionts interact and coordinate.

Increased attention has recently been paid to holobiont chronobiology and the characteristics of circadian systems in symbiotic interactions^22^, although the vast majority of investigations into the evolution and function of circadian clock components have been made in free-living organisms. To date, most holobiont chronobiology studies deal with microbiotic effects of prokaryotes and unicellular eukaryotes on host chronobiology^21,22^. Nonetheless, important inroads into the role of rhythmicity have been made in eukaryotic-eukaryotic systems ranging from arbuscular mycorrhizal fungal interactions and their host plants^26^ as well as *Symbiodinium* algae and their coral host species^23,24^. The lichen symbiosis is composed of partners with highly specialized and often well-investigated roles^49,50^, and outputs from one symbiont (such as photosynthates) may affect the production of compounds by the other symbiont^51^. Each symbiont produces compounds in a seasonally-variable manner^52,53^, which may be regulated at least in part by the photosensory and circadian machinery of the symbionts. Lichen symbioses are also remarkably stable and geographically widespread, and the exchange of symbionts along gradients has been reported (e.g.^54–56^, providing an excellent model in which to study the effect of each partner’s circadian system in the holobiont.

While we identified putative homologs of all components of the core circadian clock machinery in nearly all lineages, some of the core circadian clock genes were missing in some lineages. The gene *frq* appears to be missing from certain isolated lichen-forming fungal lineages in the Umbilicariaceae, as well as from the Trichocomaceae (*Aspergillus* spp. and *Penicillium* spp.) within the Eurotiomycetes. The main paradigm of the evolution of *frq* in the Fungi has changed considerably over the last decades. Originally identified in *Neurospora crassa* (Ascomycota, Sordariomycetes), initial investigations of its evolution restricted its presence to within the Sordariomycetes, Leotiomycetes and Dothideomycetes within the Ascomycota^46^. More recent investigations have expanded this understanding considerably, and *frq* is understood to have evolved at least before the divergence of Mucoromycotina and Zoopagomycota from Dikarya^35^.

In our present study, the putative homologs of *frq* could not be found in the genomes of certain lichen-forming fungal lineages (*U. phaea, U. deusta, U. spodochroa, U. freyi*; see Davydov et al. 2017^57^ for an overview of the Umbilicariaceae). Little life history or ecological rationale can at present be given for a parsimonious loss of the *frq* homolog in these lineages alone; interestingly, we were able to find a copy for one of these species (*U. spodochroa*) as well as for other lineages that do not have their genomes sequenced (Fig. S4), using a degenerate primer design approach (Table S2). It may be that *frq* is present in many more species but simply remains undetected in some genomes because of issues with local genome quality, genome assembly, annotation, and gene prediction. For the other circadian clock components investigated in this study, we found that *frh* and *wc-2* were occasionally missing in single disparate lineages: *wc-2* in *Alternaria alternata* and *wc-2* and *frh* in *Sordaria fimicola.* Of the four genes investigated in this study, only the gene *wc-1* was identified in every lineage without exception.

The rapid induction of *frq* expression by light has long been a characteristic phenotype of the main FWO oscillator in *N. crassa* (since e.g. Crosthwaite et al.^58^). This phenotype of light-induced *frq* expression has been observed in other Ascomycota such as the soil-living saprophyte *Pyronema confluens* (Pezizomycetes)^59^, as well as in the Mucoromycota, such as the arbuscular mycorrhizal fungus *R. irregularis*^26^. The presence of *frq, wc-1*, and *wc-2* alone, however, is not enough to infer a functional FWO oscillator: in the plant pathogen *Verticillium dahliae,* the *frq* homolog is present, but is largely unresponsive to light^60^. It is thus noteworthy that, in the present study, we observe that lichen-forming fungi from two highly diverged lineages, the Lecanoromycetes (*U. pustulata*) and the Eurotiomycetes (*D. miniatum*) both display *frq* light*-*dependent responses similar to the canonical responses of *N. crassa*, pointing to a functionally-conserved core circadian clock mechanism in at least two highly-diverged groups of lichen-forming fungi.

Circadian rhythms are central to the functioning of symbiotic interactions, as evidenced by the squid-Vibrio^25^, coral-Symbiodinium^23,24^, and plant-mycorrhiza^26^ symbioses. The lichen system is an unexplored model of symbiosis with respect to the circadian clock that has great potential in contributing to our understanding of organism-organism and organism-environment interactions. Having identified the core components of the fungal side of the lichen symbiosis and determined their functional conservation in at least two broadly-diverged lineages of lichen-forming fungi, we can now address questions like: How does the circadian system influence seasonal and diurnal dynamics in the lichen symbiosis, such as growth, nitrogen fixation, photosynthesis, or ascospore discharge? Which seasonal and diurnal rhythms are important in this mutualism in the first place? To what extent do the circadian clocks of the mycobiont and photobiont interact and interdepend? Is there circadian regulation in partner recruitment, establishment, and maintenance of the symbiotic association? What is the role of the circadian clock machinery in climatic niche specialization? An in-depth knowledge of the lichen circadian clock and its outputs and contributions to the symbiosis has the potential to enhance our understanding of symbiotic systems more broadly, from geographic distributions to interactions at the cellular and the molecular levels.

## Supplementary Information

Supplementary Information 1.

Supplementary Information 2.

Supplementary Information 3.

Supplementary Information 4.

## Data Availability

NCBI/GenBank Biosample IDs for the genomes utilized in this study published in Singh et al. 2022 (lichen/fungus): *Umbilicaria freyi*: SAMN27294873/SAMN26992773; *Umbilicaria deusta*: SAMN27294874/SAMN26992774; *Umbilicaria hispanica* (*Lasallia hispanica*): SAMN27294875/SAMN26992775; *Umbilicaria phaea*: SAMN27294876/SAMN26992776; *Umbilicaria spodochroa*: SAMN27294878/SAMN26992778; *Umbilicaria subpolyphylla*: SAMN27294879/SAMN26992779; *Umbilicaria grisea*: SAMN27294880/SAMN26992780; *Dermatocarpon miniatum*: SAMN27294881/SAMN26992781. JGI Project IDs for the genomes utilized in this study: *Sclerophora sanguinea*: 1,052,667; *Xanthoria parietina*: 16,820; *Usnea florida*: 1,051,215; *Lobaria pulmonaria*: 1,006,353; *Cladonia grayi*: Cgr/DA2myc/ss v2.0 (portal name). NCBI/GenBank Biosample IDs for all 44 remaining accession numbers/IDs of published genomes used in this study can be found in Table S1.
