# Supplementary material for: Identification and expression of functionally conserved circadian clock genes in lichen-forming fungi
Source: Sci Rep. 2022 Sep 23;12:15884. doi: 10.1038/s41598-022-19646-y (PMC9508176; doi:10.1038/s41598-022-19646-y)
Supplement: Supplementary file 1 — Supplementary Information 1. [file 41598_2022_19646_MOESM1_ESM.docx]

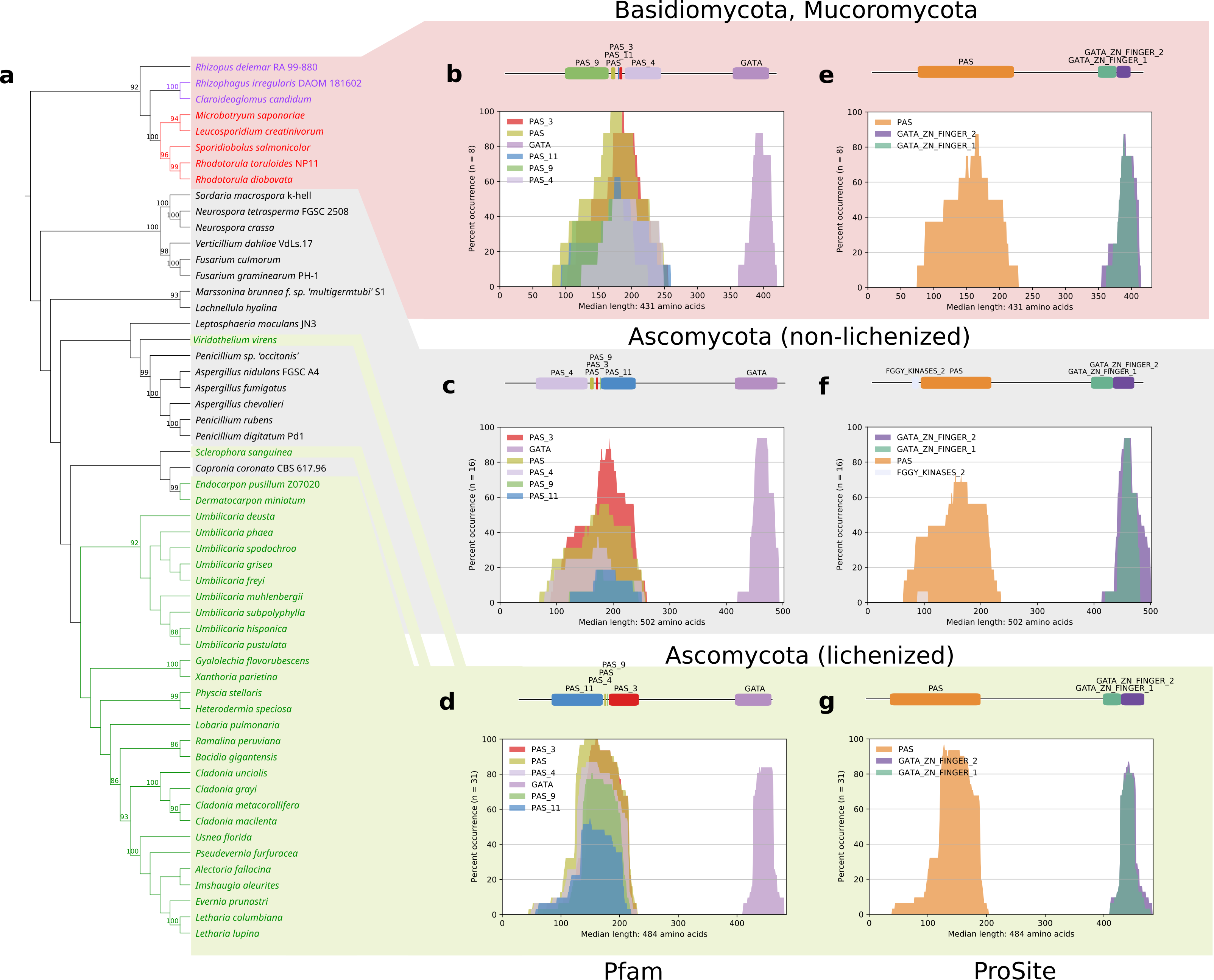
**Figure S1: Domain architecture of putative homologs of the circadian clock protein White Collar-2 is conserved in lichen-forming fungi.**

The phylogeny inferred for circadian clock homologs of WC-2 demonstrates a broad consensus with taxonomy, including for lichen-forming taxa **(a)**. Functional domains annotated according to Pfam **(b-d)** and PROSITE **(e-g)** databases in lichen-forming **(d, g)** and non-lichen-forming lineages in the Ascomycota **(c, f)** as well as the Basidiomycota **(b, e)**.


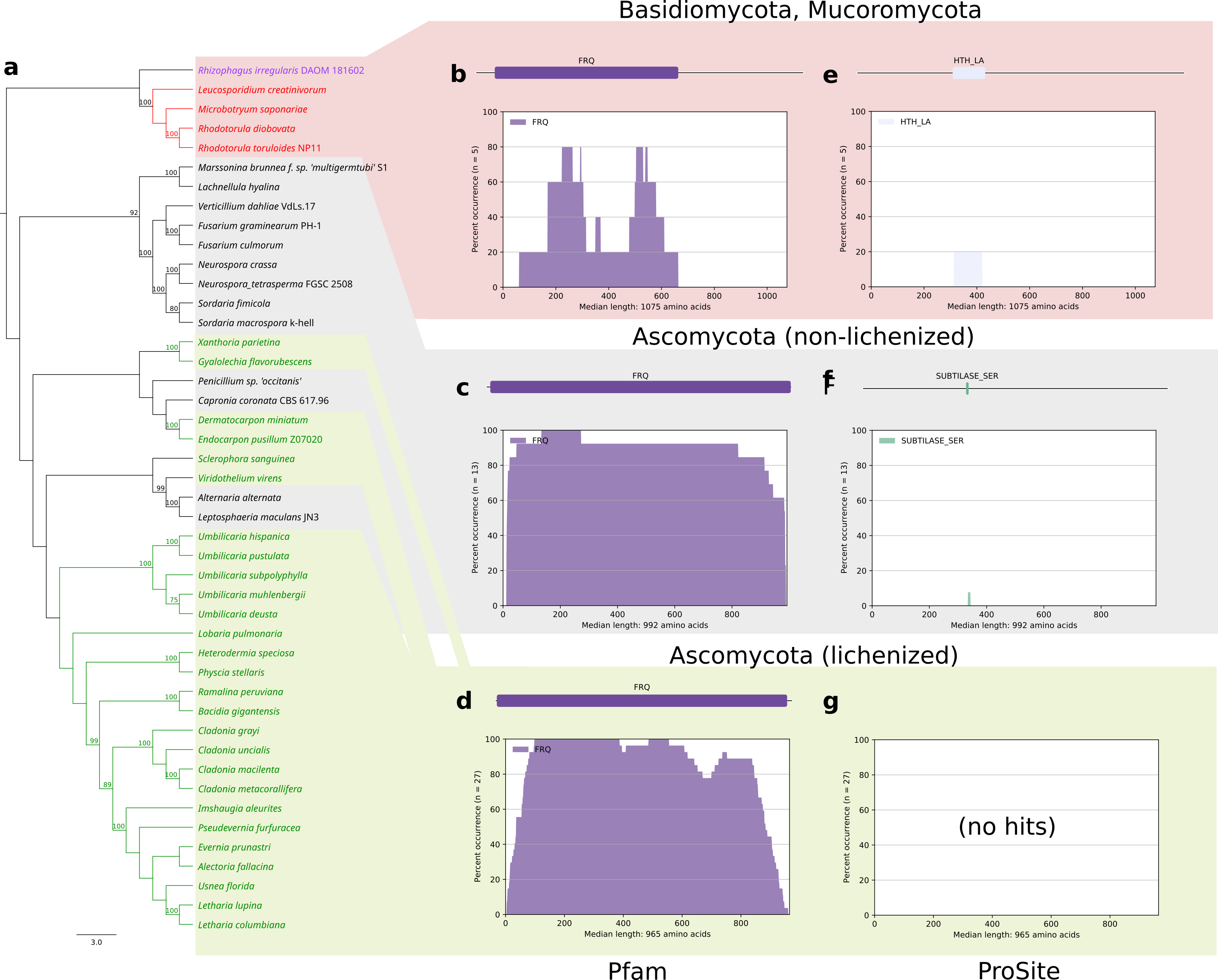
**Figure S2: Domain architecture of putative homologs of the circadian clock protein Frequency is conserved in lichen-forming fungi.**

The phylogeny inferred for circadian clock homologs of FRQ demonstrates a broad consensus with overall fungal phylogeny, including lichen-forming taxa **(a)**. Functional domains annotated according to Pfam **(b-d)** and PROSITE **(e-g)** databases in lichen-forming **(d, g)** and non-lichen-forming lineages in the Ascomycota **(c, f)** as well as the Basidiomycota and Mucoromycota **(b, e)**.


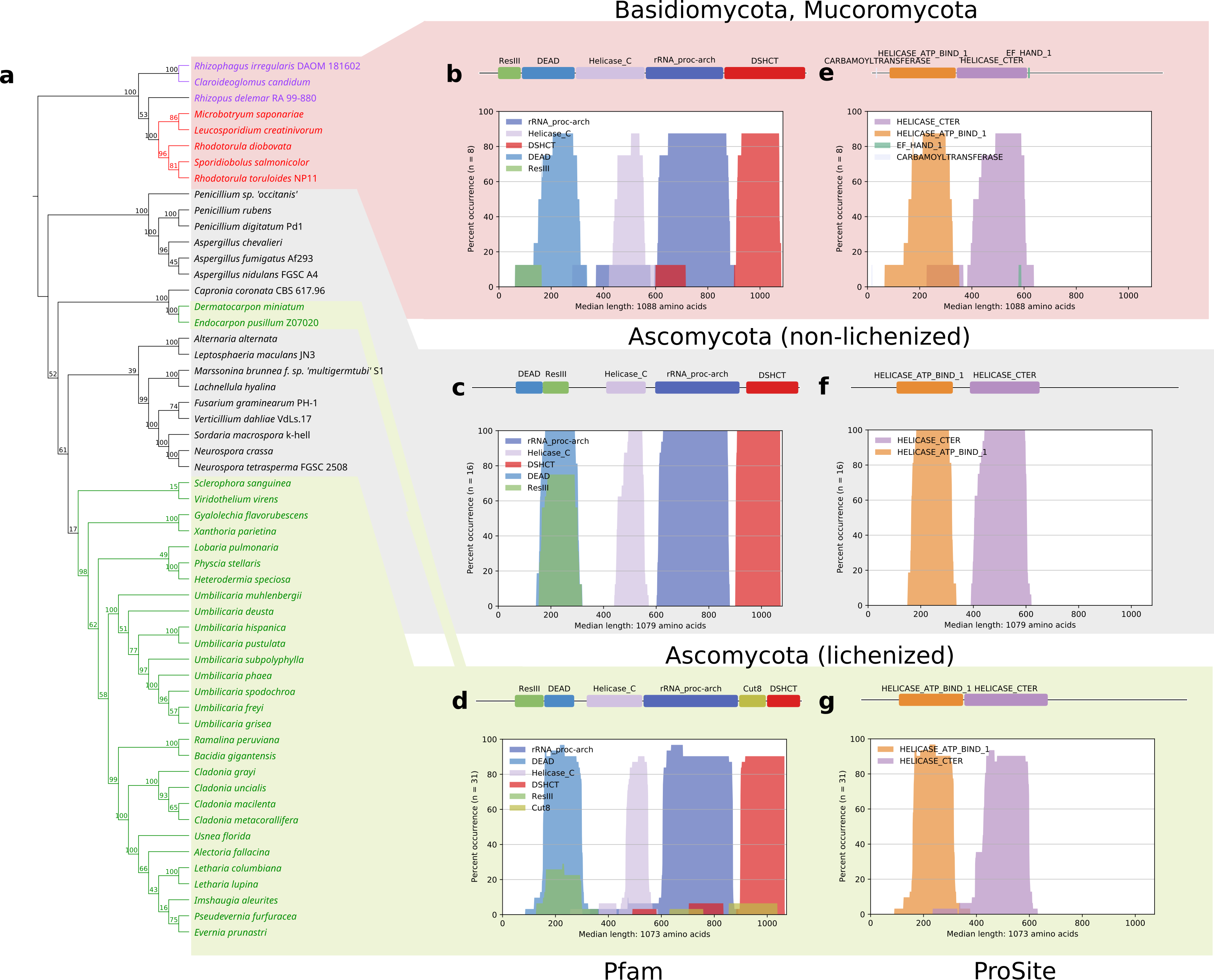
**Figure S3: Domain architecture of putative homologs of the circadian clock protein Frequency Interacting RNA Helicase is conserved in lichen-forming fungi.**

The phylogeny inferred for circadian clock homologs of FRH demonstrates a broad consensus with overall fungal phylogeny, including lichen-forming taxa **(a)**. Functional domains annotated according to Pfam **(b-d)** and PROSITE **(e-g)** databases in lichen-forming **(d,g)** and non-lichen-forming lineages in the Ascomycota **(c, f)** as well as the Basidiomycota and Mucoromycota **(b,e)**.


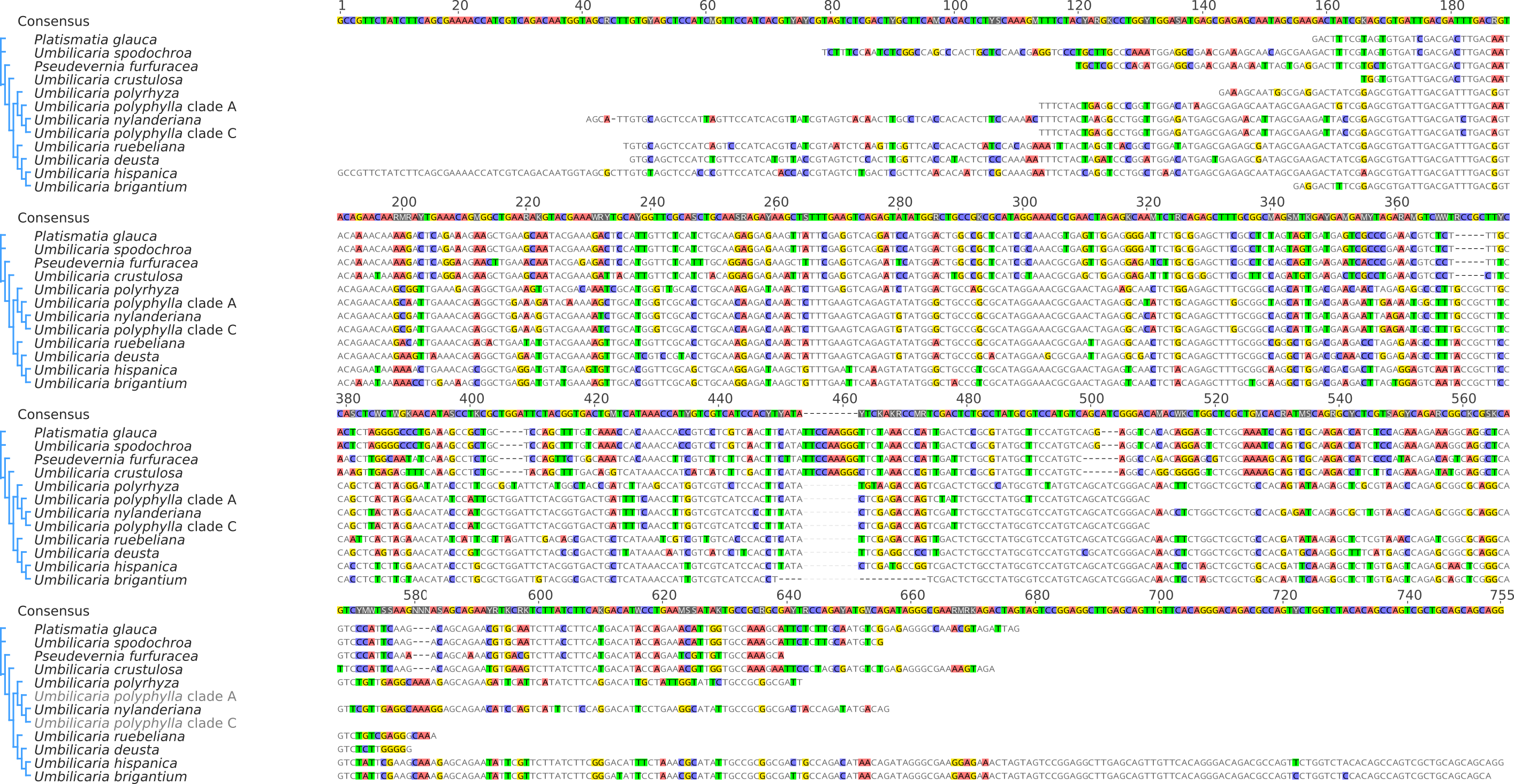
**Figure S4: Putative homologs of the circadian clock gene *frequency (frq)* identified in other lichen-forming fungi.**

Degenerate primers designed for a region of partially conserved region of of *frq* in the genomes of Lecanoromycetes spp. supports the existence of an *frq* homolog for *U. spodochroa* as well as for several other species whose genomes have not been sequenced.

**Table S1: Accession numbers and source database for the loci utilized in this study.** ^1^ Paper cited when gene set when not associated directly with GenBank/JGI accession. NCBI: National Center for Biotechnology Information; JGI: DOE Joint Genome Institute. ^2^ Biosample or Project ID for NCBI/GenBank or JGI repository, respectively. Accessions divided by a forward slash (“/”) denote the lichen (i.e. metagenomic)/fungal (i.e. mycobiont only) Biosample IDs.

**Table S2: Primers utilized both for RT-PCR (Fig. 2) and for degenerate primer-based PCR (Fig. S4).**

**Table S3: Statistical results for RT-PCR experiments in Fig. 2.**
